# Restoration of biogeomorphic systems by creating windows of opportunity to support natural establishment processes

**DOI:** 10.1002/eap.2333

**Published:** 2021-05-07

**Authors:** Gregory S. Fivash, Ralph J. M. Temmink, Manuel D’Angelo, Jeroen van Dalen, Wouter Lengkeek, Karin Didderen, Francesco Ballio, Tjisse van der Heide, Tjeerd J. Bouma

**Affiliations:** ^1^ Department of Estuarine and Delta Systems Royal Netherlands Institute for Sea Research Korringaweg 7 Yerseke 4401 NT the Netherlands; ^2^ Groningen Institute for Evolutionary Life Sciences Community and Conservation Ecology Group University of Groningen Nijenborgh 7 Groningen 9747 AG the Netherlands; ^3^ Aquatic Ecology and Environmental Biology Institute for Water and Wetland Research Radboud University Heyendaalseweg 135 Nijmegen 6525 AJ the Netherlands; ^4^ Department of Civil and Environmental Engineering Politecnico di Milano Piazza Leonardo da Vinci 32 Milano 20133 Italy; ^5^ Bureau Waardenburg Varkensmarkt 9 Culemborg 4101 CK the Netherlands; ^6^ Department of Coastal Systems Royal Netherlands Institute for Sea Research Landsdiep 4 't Horntje (Texel) 1797 SZ the Netherlands; ^7^ Department of Physical Geography Faculty of Geosciences Utrecht University Princetonlaan 8a Utrecht 3584 CB the Netherlands

**Keywords:** biogeomorphic systems, microtopography, natural establishment, restoration, salt marshes, windows of opportunity

## Abstract

In degraded landscapes, recolonization by pioneer vegetation is often halted by the presence of persistent environmental stress. When natural expansion does occur, it is commonly due to the momentary alleviation of a key environmental variable previously limiting new growth. Thus, studying the circumstances in which expansion occurs can inspire new restoration techniques, wherein vegetation establishment is provoked by emulating natural events through artificial means. Using the salt‐marsh pioneer zone on tidal flats as a biogeomorphic model system, we explore how locally raised sediment bed forms, which are the result of natural (bio)geomorphic processes, enhance seedling establishment in an observational study. We then conduct a manipulative experiment designed to emulate these facilitative conditions in order to enable establishment on an uncolonized tidal flat. Here, we attempt to generate raised growth‐promoting sediment bed forms using porous artificial structures. Flume experiments demonstrate how these structures produce a sheltered hydrodynamic environment in which suspended sediment and seeds preferentially settle. The application of these structures in the field led to the formation of stable, raised sediment platforms and the spontaneous recruitment of salt‐marsh pioneers in the following growing season. These recruits were composed primarily of the annual pioneering *Salicornia* genus, with densities of up to 140 individuals/m^2^ within the structures, a 60‐fold increase over ambient densities. Lower abundances of five other perennial species were found within structures that did not appear elsewhere in the pioneer zone. Furthermore, recruits grew to be on average three times greater in mass inside of the structures than in the neighboring ambient environment. The success of this restoration design may be attributed to the combination of three factors: (1) enhanced seed retention, (2) suppressed mortality, and (3) accelerated growth rates on the elevated surfaces generated by the artificial structures. We argue that restoration approaches similar to the one shown here, wherein the conditions for natural establishment are actively mimicked to promote vegetation development, may serve as promising tools in many biogeomorphic ecosystems, ranging from coastal to arid ecosystems.

## Introduction

Biogeomorphic landscapes are unified by the interaction between dynamic physical transport processes and the mediation of these processes by biological mechanisms (Corenblit et al. 2011). This classification spans a diverse range of vegetated coastal systems such as dunes, seagrass meadows, salt marshes, and mangrove forests, as well as many physically driven terrestrial and aquatic systems from arid landscapes to riparian zones (Corenblit and Steiger 2009). In each of these cases, vegetation plays a major role in the physical stabilization of the landscape. In these systems, the transport of sediment, either through the flow of air or water, is passively suppressed and redirected by the presence of vegetation (Temmerman et al. 2007, Murray et al. 2008). The landscape stabilization driven by the presence of vegetation feeds back upon itself by making the environment more hospitable to further vegetation, which provokes further landscape development and stabilization. However, when vegetation is initially absent the predominance of physical drivers may create an environment that is nearly uninhabitable to pioneering species. The self‐reinforcing nature of this stabilization process encourages the system to maintain whatever is its current condition, be it bare or fully vegetated (Moffett et al. 2015). Only extreme events have the potential to shift the system between vegetated and unvegetated states. To date, this characteristic “alternative stable state” behavior has been independently demonstrated to occur in seagrass meadows (van der Heide et al. 2007), rivers (Heffernan 2008, Wang et al. 2016), and salt marshes (Wang and Temmerman 2013), among many others (Schröder et al. 2005). Until recently, studies on alternative stable state systems have predominantly focused on predicting how extreme disturbance events may lead to a shift in ecosystem state from vegetated to degraded that is extremely difficult to reverse (Beisner et al. 2003, Suding et al. 2004). Yet there also exists a range of conditions on the opposing end of the spectrum that allows for a drastic natural shift toward a vegetated state (see Holmgren and Scheffer 2001 for one example). The focus of this study is to demonstrate how an understanding of these “establishment” dynamics may be used to perform landscape restoration.

In general, the circumstances under which an individual pioneer may pass from the seed stage to a developed and well‐established adult plant capable of influencing its physical environment are rare in dynamic biogeomorphic landscapes (Balke et al. 2014). In order for new recruits to establish, the most vulnerable phases of the life‐cycle must coincide with circumstances where, for a time, environmental disturbances are exceptionally benign: a so‐called “window of opportunity” (Balke et al. 2011, Hu et al. 2015). As time passes under tolerable conditions, recruits gain the capacity to survive under increasingly harsh conditions because of the development of protective structures (e.g., stiff stems, long roots, Cao et al. 2018). Furthermore, as the patch size or shoot density of an organism increases, modifications on the environment also increase survival odds (Bouma et al. 2009, Friess et al. 2012, Katwijk et al. 2016). This results in a pattern of infrequent, event‐driven establishment behavior in these systems (Balke et al. 2014).

Biogeomorphic systems have experienced worldwide degradation as a consequence of human impacts, which have accelerated in recent decades (Lotze et al. 2006). The loss of these systems has, in turn, had negative consequences for local populations in terms of flood safety, arable land, and water storage to name a few (Costanza et al. 1997, Le Maitre et al. 2007, Barbier et al. 2011, Temmerman et al. 2013). Because of the absence of crucial natural infrastructure in unvegetated biogeomorphic environments, natural recolonization does not readily take place. Thus, there is a desire for the development of active means by which to revegetate these degraded landscapes. Restoration in recent decades has focused strongly on the seeding of barren areas and transplantation of adult organisms (Bainbridge et al. 1995, Bean et al. 2004, Abella and Newton 2009, de Groot and van Duin 2013, Mor‐Mussery et al. 2013, Han et al. 2015). However, in biogeomorphic systems this practice has been demonstrated to be significantly less effective than in other environments (Beck and Airoldi 2007). For instance, in salt‐marsh and mangrove systems, past studies have found that seed retention rather than seed availability plays the crucial limiting role in seedling establishment (Balke et al. 2011, Zhu et al. 2014, Wang et al. 2019). Similarly, in salt‐marsh systems, the appearance of young yearling recruits in the pioneer zone often fails to amount to permanent marsh expansion because of extremely poor winter survival of yearling plants (Balke et al. 2014). Furthermore, the survival of transplants in the first year is commonly below 50% among restoration projects in disturbance‐driven coastal (Bergin 1994, Bayraktarov et al. 2015, Silliman et al. 2015, Derksen‐Hooijberg et al. 2018) and arid environments (Bainbridge et al. 1995, Bean et al. 2004, Abella and Newton 2009, Mor‐Mussery et al. 2013).

Instead of attempting to understand and take advantage of the natural dynamics of these systems, the direct application of plant material remains the primary method for revegetation due to historical precedent in forestry (Bainbridge et al. 1995) and ignorance of alternative options (Silliman et al. 2015). These active measures tend not to reflect natural mechanisms of colonization in biogeomorphic systems, which depend on the momentary alleviation of hostile abiotic conditions (Balke et al. 2011, Hu et al. 2015). If efforts could instead be re‐focused upon achieving short‐term manipulation of abiotic variables, it may be possible to manifest establishment events in these systems artificially (Balke et al. 2014).

One strong candidate abiotic variable with which to manipulate seedling establishment may be found in the pioneer zone of salt marshes. Here, raised sediment surfaces are reported to have growth‐amplifying effects on young salt‐marsh recruits (Fivash et al. 2020, Mossman et al. 2020). This may be due to special conditions within raised sediment platforms, from which water can easily drain over the low‐tide interval (Fagherazzi and Mariotti 2012). Lab experiments have demonstrated that the well‐drained sediment pore spaces within raised sediment surfaces experience better recirculation by tidal flows (Fivash et al. 2020). The enhanced recirculation increases oxygen penetration and likely reduces the presence of sulphidic soil toxins (Koch and Mendelssohn 1989, Lamers et al. 2013). To date, however, the evidence of the relationship between raised sediment surfaces and pioneer establishment has been most commonly cited using anecdotal evidence and a few recent manipulative field experiments (Xie et al. 2018, Mossman et al. 2020). The potentially major and widespread role that pioneer zone topography may play in the natural expansion of salt marshes has yet to be documented in relation to naturally occurring mudflat heterogeneity. Thus, we attempt briefly to preface our experimental work here with an observational survey that demonstrates the effect that such natural bed forms have on seedling establishment.

Such bed‐form heterogeneity is quite common in nature and can be generated by either physical processes such as creek incision (Temmerman et al. 2007), through biogeomorphic feedbacks driven by algal mats (Blanchard et al. 2000, van de Vijsel et al. 2020), or as a consequence of the pioneering vegetation itself (Bouma et al. 2009). Earlier studies have also shown that artificial epibenthic structures have the capacity to generate similar microtopography by reproducing the altered flow patterns within and around pioneer salt‐marsh patches (Bouma et al. 2007). Such vegetation patches, typified by the clonal pioneer *Spartina anglica*, commonly generate raised sediment mounds internally (Bouma et al. 2009). The first study to mimic the effects of pioneer vegetation in the field demonstrably used a network of bamboo poles to approximate stiff aboveground shoots on a mudflat (Bouma et al. 2007). The bamboo poles modified the flow dynamics within and around the patch, producing an altered sediment bed morphology characteristic of pioneer patches.

In this study we attempt to create conditions favorable for natural establishment of salt‐marsh seedlings using artificial structures to alter the abiotic environment by creating raised sediment mounds in the pioneer zone. Prior to a field test, we (1) perform an observational survey on a salt‐marsh pioneer zone to measure the effect that naturally occurring microtopographic heterogeneity has on establishment success. These data then act as a baseline with which we can compare the results of the restoration experiment. Next, (2) we test the artificial structure’s role in modifying flow patterns and concentrating seeds by subjecting it to a series of flume tests, relative to a control. Finally (3), in a field experiment, 18 structures are installed on the mudflat adjacent to a degraded salt marsh where the topographic development and recruitment of salt‐marsh plants is then monitored over 2 yr, and contrasted against recruitment in the unmodified pioneer zone.

## Materials and Methods

### Section 1: Observational survey

#### Observational study—Field site

**Fig. 1:**
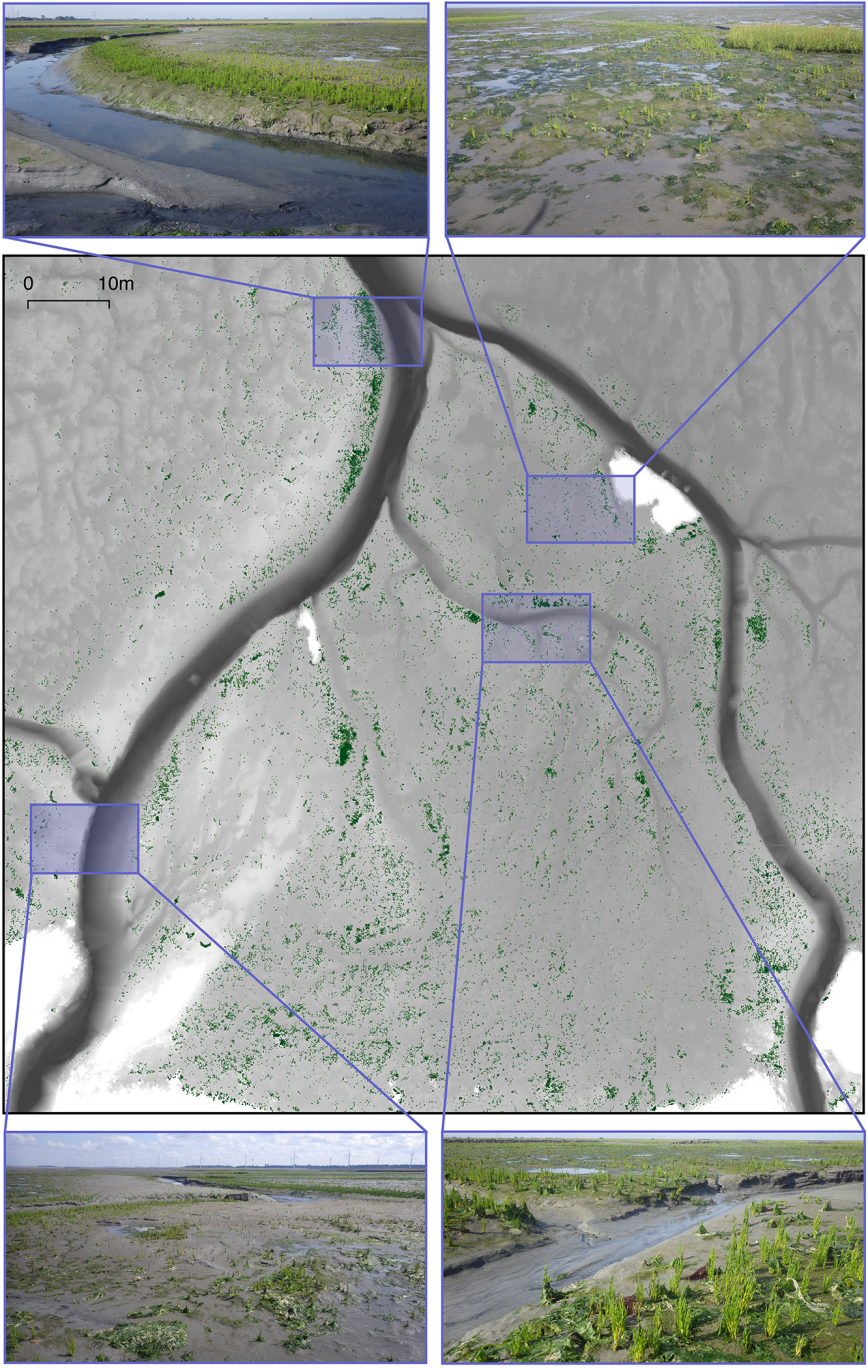
A map of the observational study site at Rattekaai, in the Eastern Scheldt of the Netherlands. Gray tones from light to dark in the central frame indicate increasing inundation frequency. Green pixels denote the presence of vegetation as classified from laser scan data. Panel photographs (top and bottom) give an indication of the appearance of the study site. The four blue rectangles refer to the areas of the site encapsulated in the photos.

We prefaced our experimental study by conducting an observational survey to demonstrate the supportive role of raised sediment microtopography on salt‐marsh pioneer establishment. This survey was performed on the pioneer zone of the salt marsh Rattekai in the Eastern Scheldt estuary of the Netherlands (51.441643, 4.196388). This site was chosen for its topographic variability and strong recruitment of *Salicornia* *procumbens* over the growing season of 2019 (Fig. 1). Where these two characteristics occurred together it allowed for the ecological benefits of these topographic patterns to be measurable in the variation in the density of the annual recruits (assuming that seed availability was not a constraint).

#### Observational study—Laser scan survey

**Fig. 2:**
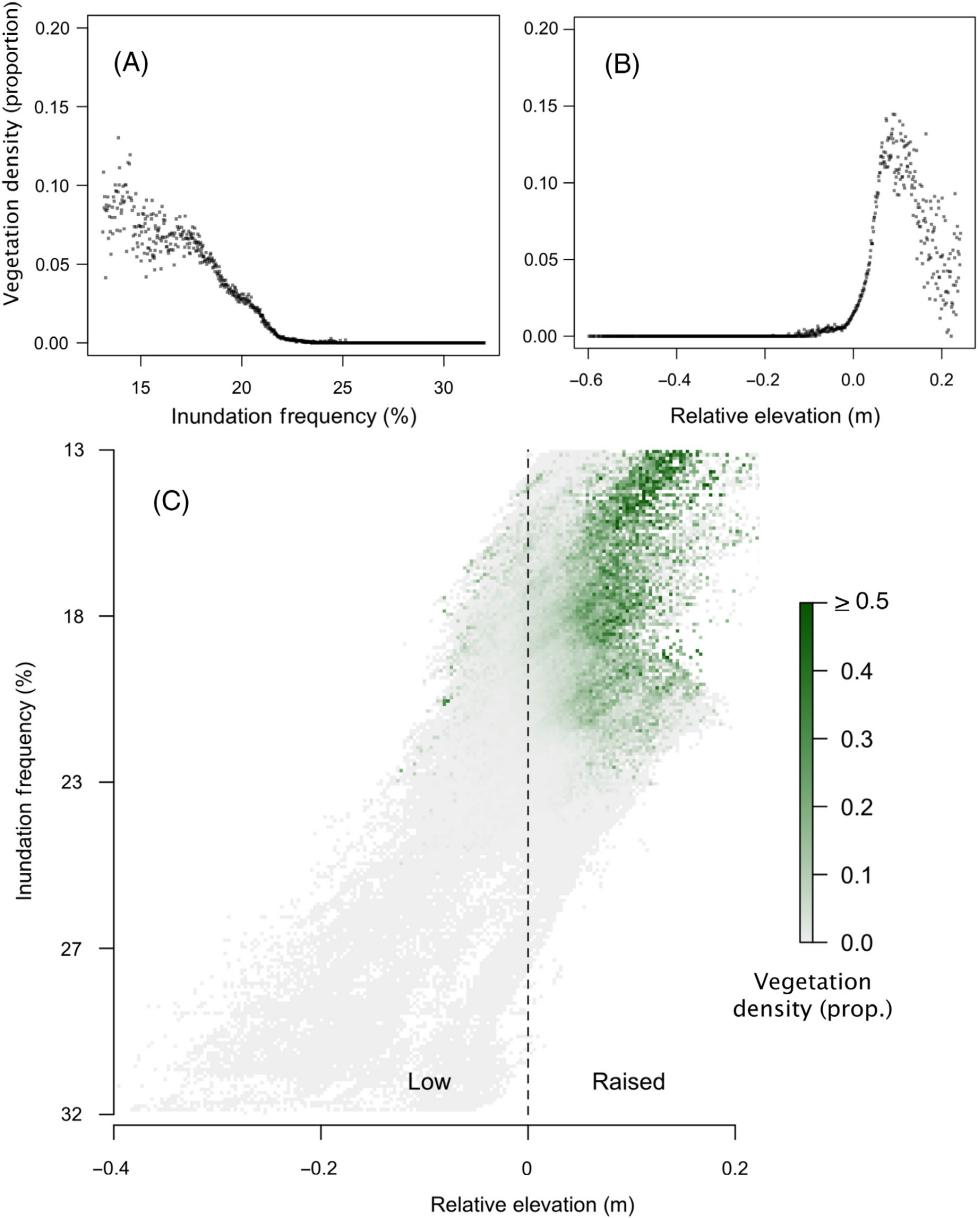
Correlative analysis displays how vegetation density varies depending on both inundation frequency and relative elevation. First (A, B) correlations are displayed considering only one of the two variables at a time. (C) A two‐dimensional density plot then demonstrates the disentangled effects of inundation frequency and relative elevation. Both low inundation frequency and raised relative elevation appear to be required for vegetation establishment to occur.

In July 2019 we scanned the bathymetry of the mudflat using a terrestrial laser scanner (*RIEGL* VZ‐400i, *RIEGL* Laser Measurement Systems GmbH, Horn, Austria). In total, four scans were combined to map a 12,100‐m^2^ area (1.21 ha). The resultant point cloud (containing >50 million points) was then used to generate a raster of elevation values at 0.01‐m^2^ resolution. The absolute elevation of the mudflat was then used to calculate the inundation frequency as well as the “relative” elevation of each raster pixel. The absolute elevation was calculated using the 30% quantile of the raw point cloud (instead of the mean or median) to avoid allowing vegetation in the landscape to raise the mudflat elevation incorrectly. Other features such as *S*. *anglica* patches and rock wall features were also explicitly excluded from the analysis by removing pixels above a threshold elevation value. The elevation raster of the study area was converted to an inundation frequency map based on publicly available water‐level time series data collected by Rijkswaterstaat (The Dutch Ministry of Infrastructure and Water Management) at the nearest monitoring point, Marollegat station. The “relative” elevation (a measure of the microtopography in the landscape) was calculated by comparing the elevation of each pixel with the average elevation value of a sampled square area around that point. The results displayed here use a 2 × 2 m sample grid (Fig. 2); however, the correlation between relative elevation and vegetation density was also tested for a spectrum of grid sizes between 0.1 and 10 m (Appendix S1: Fig. S1). The minimum neighborhood length of 0.1 m is the smallest possible for our grid resolution, and at the maximum neighborhood of 10 m the window size is so large that the response of the relative elevation becomes very similar to that of inundation frequency, characterizing large‐scale elevation differences rather than microtopography.

#### Observational study—Vegetation classification

*Salicornia* vegetation was identified within the laser scan point cloud using a technique similar to that described in Sharma et al. (2010). Here, *Salicornia* individuals appeared as vertical stacks of pixels above the mudflat, which could be differentiated from the bed by the high slope relationships between themselves and neighboring pixels. Vegetation classification was performed on 10 × 10 m sample grids in which a point was labeled as vegetation when the average slope between it and the other points in the sample grid exceeded an average of 0.027 radians. The lowest 10% of points were excluded from consideration as vegetation (see Appendix S1: Fig. S2 for visualization of this method). The application of this approach was supervised and adjusted where necessary to avoid false‐classification of mudflat features with high slope such as creek banks. A 0.01‐m^2^ raster for vegetation density was then created by calculating the proportion of the total points labeled as vegetation within each raster pixel (thus constrained between 0 and 1). We then were able to pair the vegetation density map with the bathymetric maps to seek patterns in establishment in response to variation in both relative elevation and the inundation duration over the entire study area. R scripts and data used to perform all analysis detailed here are available within 4TU.Research Data.

#### Observational study—Ground‐truthing survey for pioneer occurrence

In addition to conducting laser scans, we also performed a ground‐truthing survey to give a more accurate description of the pioneer vegetation on a more limited scale. Over an evenly spaced grid of 126 points, which spanned the scanned area, we sampled the pioneer vegetation within a 0.25‐m^2^ quadrat. The maximum height of the central stem was measured of every *Salicornia* individual within the quadrat. One hundred individuals were selected at random to act as representatives in a biomass calibration between the height of the central stem and the individual dry biomass. In addition to height measurements, these individuals were dried at 60°C to constant mass and then weighed. A calibration between dry mass and central stem height was used to calculate the total biomass density (in grams per square meter) within each quadrat (*R* ^2^ = 0.873, *n* = 97, *P* < 0.0001; Appendix S1: Fig. S3). When length measurements of *Salicronia* individuals were later made in our experimental study, the same biomass calibration was used to estimate their mass. In this way, measurements could be used to draw comparisons of the recruit size between our observational and experimental studies.

### Section 2: Flume study

#### Artificial structure

We then sought to recreate the environment present artificially on raised sediment surfaces present in our observational survey to induce the establishment of recruits in other areas. Our approach was inspired by the flow reduction patterns present within pioneering salt‐marsh grasses such as *S. anglica*, which produce raised mounds internally. The same process of flow manipulation was attempted here using artificial structures. These structures, based essentially on the features of an earlier bamboo mimic, which recreated the flow conditions within clonal patches of the salt‐marsh pioneer, *Spartina* *angelica* (Bouma et al. 2007), are made of a complex lattice that limits flow as a consequence of its shape, but allows for the passage of water and sediment. The structures of concern here, known as BESE‐elements (BESE, Culemborg, the Netherlands; Appendix S1: Fig. S4), are made of biodegradable potato‐waste‐derived Solanyl C1104M (Rodenburg Biopolymers, Oosterhout, the Netherlands) and are constructed by stacking separate repeating layers to form each unit (three layers in this study). The surface‐to‐volume ratio of the structure is 80 m^2^/m^3^ with a pore size of 40 mm (further information concerning this structure can be found in Temmink et al. 2020).

#### Flume study—Racetrack flume experiments

**Fig. 3:**
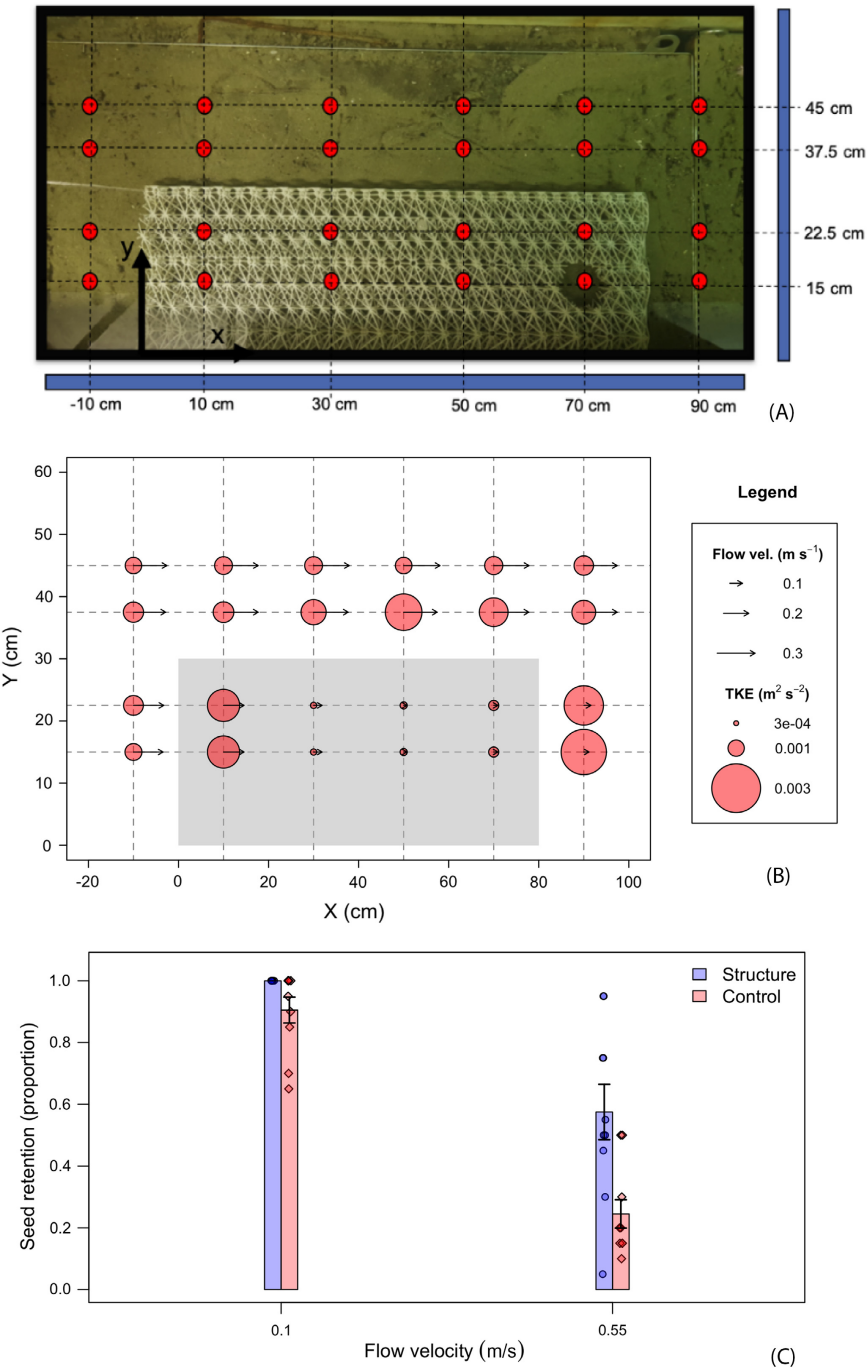
(A) Schematic of the flume experimental setup. In hydrodynamic tests (B), turbulent flows generated within the structure dissipate energy to reduce flow velocity, which in turn (C) reduces the probability of seed resuspension within the structure. Both average flow velocity and turbulent kinetic energy (TKE) values displayed here are calculated from a measurement volume 3.4 cm in height, centered 3 cm above the bed (see text for details).

We performed a series of flume tests to gauge our experimental structure’s capacity to modify the flow of water in order to facilitate the accretion of sediment and seeds in the field. In the first of these tests (1) we measured the modification of flow and turbulence around and within the structure. We then (2) measured the capacity for the low‐flow zone within the structure to preferentially accumulate seeds with seed retention tests. All flume tests were performed in the racetrack flume at the Netherlands Institute for Sea Research in Yerseke (van Wesenbeeck et al. 2008). This flume is composed of two parallel 10.8‐m channels of 0.6‐m cross‐section width connected at either end so that water may flow in a continuous cyclical path through the flume. The experimental section of the flume is a 2‐m section at the end of the channel opposite where the flow modifications are performed in order to allow for turbulence produced during acceleration to disperse and for flow to become laminar before reaching the experimental region. In this opposite channel, flow is accelerated up to a specified velocity using a paddle wheel. An artificial 30 × 80 × 6 cm structure was placed across half of the flume channel width and secured to the experimental area with a fine sediment bottom using anchoring pins (Fig. 3A). With this design it was possible to measure both flow attenuation within the structure and flow acceleration around the structure (as seen also in Bouma et al. 2007). The flume was then filled with seawater from the Eastern Scheldt (salinity 32 ppt) to a water height of 35 cm.

#### Flume study—Effects of structure on hydrodynamics

In the first of these experiments, the flow velocity in the flume was accelerated to approximate the range of conditions typical of tidal flows over intertidal zones in the Dutch Scheldt estuaries. These chosen flow velocities, 0.085, 0.145, and 0.23 m/s, were based on published data on tidal flow in the Western Scheldt to represent typical high‐tide flow velocities experienced at spring, mid, and neap‐tide (Bouma et al. 2005). The same procedure was duplicated in each experiment when the structure was absent to act as a control.

Water velocity was measured in three dimensions using a Vectrino Profiler (Nortek AS, Oslo, Norway), an acoustic Doppler velocimeter (ADV), over a coordinate volume of 72 points (see Fig. 3A). For those points that coincided with regions within the structure, a 7‐cm‐diameter cylindrical hole was cut into the structure so that the ADV could be placed safely within the structure. In this way, measurements could be performed with the minimum of refraction off of the structure, while also attempting to limit the influence of the hole on flow dynamics. One additional measurement was taken upstream in the flow channel to use as a baseline for flow velocity in each experimental treatment. Each measurement took place over a sampling interval of 3 min at a frequency of 100 Hz, yielding in total of 18,000 instantaneous flow velocity values per measurement, averaged amongst a 3.4‐cm sampling volume discretized between 18 0.2‐cm cells. These measurements were filtered using generated SNR (signal‐noise ratio) and correlation values generated by the ADV (SNR > 25 dB, correlation >90%). As a consequence, the analysis was usually limited to the centermost 3–12 cells, because of typical inaccuracies caused by beam divergence of the ADV (Lhermitte and Serafin 1984, Zedel et al. 1996). This time series was used to calculate a single average velocity value at each of the 72 positions in the principle direction of flow (x). From this measurement volume, our analysis focused on the lowest measured sampling volume, centered 3 cm above the bed. Turbulent kinetic energy (TKE) values were also derived from the fluctuations in the three‐dimensional velocity time series data around the mean values according to in equation below. Here, *u*', *v*', and, *w*' represent the variance of the flow velocity around the mean in the *x*, *y*, and *z* domains (Stapleton and Huntley 1995):

$$\text{TKE =}\;0.5\times \left({\left(u^{\prime }\right)}^{2}+{\left(v^{\prime }\right)}^{2}+{\left(w^{\prime }\right)}^{2}\right).$$

#### Flume study—Effects of structure on seed retention

Next we performed a flume experiment to measure the seed retention capacity in regions under the influence of the structure. In this experiment, we chose to use *S. anglica* seeds as a general representative for dispersing salt‐marsh seeds. In our seed retention tests, groups of 20 seeds were placed randomly underneath a structure, or in the case of the controls, in the area the structure would have occupied. We then applied a moderate (10 cm/s) and an extreme (55 cm/s) flow environment within the channel for an interval of 5 min and recounted the seeds remaining in the experimental zone. The 55‐cm/s flow represented the highest safely achievable flow by this flume. Each treatment was repeated 10 times, each time with 20 initial seeds. Differences in the proportion of seeds retained on the sediment bed were contrasted between those protected by the structure and a control environment with generalized linear models using quasibinomial errors. Comparative tests were performed independently within each hydrodynamic environment.

### Section 3: Field experiment

#### Field study—Experimental site

Our field experiment was performed near the salt marsh “De Schorren” on the northeast coast of Texel, the largest barrier island in the Dutch Wadden Sea. The Wadden Sea is a 10,000 km^2^ estuary situated at the interface between the North Sea and the coasts of Germany, Denmark, and the Netherlands. It is a mesotidal estuary with a characteristic tidal amplitude of about 2 m. Salinity in the Wadden Sea varies tidally and seasonally between 24 and 32 ppt (van Aken 2008) and flow velocity over the mudflat can vary between 0 and 0.4 m/s over the tidal interval (Janssen‐Stelder 2000). Large mudflats with low slope in a mesotidal system make for large potentially habitable regions for salt marshes. The mudflat on this site was selected for its lack on naturally occurring microtopographic features. In contrast to many commonly studied U.S. salt‐marsh sites, the sediment characteristics of the island sites in the Dutch Wadden Sea reduce the tendency of sulphide production as a consequence of the large grain size and lower organic matter content. Sediment in this area is composed of highly mineral sand–silt mixtures (D50: 145.59 ± 1.14 μm, silt: 21.17 ± 0.3%, organic C: 0.5 ± 0.01%). Three months of pressure measurements during the winter between December 2017 and February 2018 across our experimental region indicated an inundation frequency of 20 ± 1.7%. The mean water depth during high‐tide intervals was 26 ± 0.05 cm, with a maximum recorded water height of 147 cm at spring high tide. Spectral analysis of waves suggested overall sheltered conditions of an average significant wave height of 1.34 ± 0.04 cm. The sensors were designed and constructed by the Royal Netherlands Institute for Sea Research (NIOZ Texel, the Netherlands).

#### Field study—Experimental design

In May 2016, at the southern end of this marsh along a 2‐km length parallel to the embankment, we set up an experiment using 1‐m^2^ artificial structures, 6 cm in height. In total, we placed 18 structures and designated 32 control plots in the pioneer zone. All plots were positioned approximately 30 m from the salt‐marsh margin, with a minimum of 3 m between each plot, to limit the influence of each structure on the others. Control plots were 1‐m^2^ square areas of native mudflat marked out using bamboo poles, but otherwise left unaltered. The experiment remained in place for two growing seasons until removal during the winter of 2018.

#### Field study—Sediment bed level measurements and characterization

The relative sediment bed level was measured to track local changes in accretion and erosion within and around the structures, compared against unmodified controls. At half of the structures and control plots we placed a pair of transects covering both the central 1‐m region within the structure and two 1‐m sections on either side, first perpendicular and then parallel to the shoreline. The relative sediment height (±0.1 cm) was measured over the two 3‐m transects at intervals of 5–10 cm with respect to a leveled ruler set between two permanently installed PVC reference poles. Sediment bed level changes were measured at 1‐month intervals early in the experiment, but with diminishing frequency as the sediment level modified by the structures reached an equilibrium state. The slope of the mudflat was removed from bed level measurements by performing a linear detrend over each transect. At the conclusion of the experiment sediment samples were taken to 3‐cm depth using a 2.5‐cm‐diameter open‐ended syringe from both inside of the structures and control plots. From these samples, the silt content and median grain size was determined through grain size analysis (Mastersizer 2000; Malvern Panalytical Ltd., Malvern, UK) and organic carbon and nitrogen content was determined with elemental analysis (Carlo Erba NA‐1500; Thermo Scientific, Waltham, Massachusetts, USA). All data fit the assumptions of normality and homogeneity of variance so that the sediment character and bed level within the artificial structures could be compared with controls through one‐way ANOVA tests.

#### Field study—Monitoring of natural recruitment

In 2017 we monitored the development of spontaneously occurring recruitment within the structures and on the surrounding mudflat. Monitoring was conducted first in July and repeated in September to investigate growth and mortality over the growing season. Individual densities were recorded inside of the 1‐m^2^ structures and contrasted against the control plots as well as in four 1‐m^2^ quadrats directly adjacent to each plot in each cardinal direction. Seedling individual densities were contrasted between the structure and in the ambient environment characterized by both control plots and the four adjacent sampling locations, yielding 18 replicates within the structures and 236 control plots. It was possible to group the adjacent sampling plots and the central control plots after proving them to be statistically similar. Recruits were identified to the genus level and the species level when possible. Individual densities were contrasted between these environments using a generalized linear model with quasipoisson errors.

Within each quadrant the maximum height of recruits was sampled and measured to 0.1‐cm accuracy. In the case of *Salicornia* spp., the most commonly appearing taxa in our experiment, a calibration was performed to convert between maximum central shoot height (centimeters) and total dry biomass (milligrams). The same calibration applied to the central stem length of individuals in the observational study was repeated to derive the individual mass of recruits in this experiment. In order to test for changes in density due to mortality between July and September, the date and the interaction between the structure variable and the date were also included as explanatory variables in this model. Individual biomass data were log‐transformed and then contrasted between control and structure plots using a two‐way ANOVA, similarly considering both the structure treatment and date.

### Comparison of recruitment between observational and experimental studies

Lastly, a comparison was made between the average individual size and the individual density of recruits between the observational survey on Rattekaai and the experimental study on Texel. Individual recruits sampled in the month of July (although in different years: 2017 and 2019) were grouped into control and structure groups on Texel, and the observational study on Rattekaai. The individual mass of individuals three groups was log transformed and compared via one‐way ANOVA. Individual densities were likewise compared using a generalized linear model with quasipoisson errors. All analyses were performed in R (R Development Core Team 2008).

## Results

### Observational study

Our observational study clearly demonstrates the role of both large‐ and small‐scale bathymetric features in facilitating the occurrence of *Salicornia* *procumbens*. Firstly, the appearance of *Salicornia* appeared to be constrained within a specific interval of the tidal frame. In our study site, *Salicornia* did not occur below an inundation frequency of 25% (Fig. 2A). Within this range, the *Salicornia* were further limited to areas of raised relative elevation, appearing only very rarely in areas that were on average lower than the surrounding mudflat, represented by a relative elevation <0 m (Fig. 2B). Vegetation remained largely constrained to areas of raised relative elevation, regardless of the scale at which relative elevation is measured (Appendix S1: Fig. S1). By separating the contribution of the inundation frequency and relative elevation (visualized in Fig. 2C), it can be seen that, although these two components of the landscape are correlated, they appear to both contribute independently to the density of seedling establishment. Ultimately, wherever either of the two components did not fall within its threshold range, vegetation largely ceased to occur.

### Flume study—Effects of structure on hydrodynamics

Flume experiments measuring the effect of the artificial structures on flow patterns demonstrate that flow velocities are reduced by an order of magnitude over the first 50 cm of the 80‐cm‐long structure (from 8.5, 14.5, and 23 cm/s to 0.7, 1.6, and 2.5 cm/s). This effect was dimensionally consistent over the tested range of flow velocities, consistently reducing flow by one order of magnitude over the length of the structure. Flow also accelerated slightly to the sides of the structure with respect to the incoming flow (up to 25.9 cm/s beside the structure and in the case of 23 cm/s flow). During flow deceleration in the foremost section of the structure, energy was dissipated in turbulent flows very rapidly creating a high‐stress environment. In the foremost 10 cm of the structure, turbulent kinetic energy values reached as high as 0.002 m^2^/s^2^ under 23 cm/s flow. However, this turbulent region dissipated quickly over the length of the structure, leading to a low‐turbulence, low‐flow environment in the remaining section of the structure (an average of 4.8 ± 0.2 × 10^−4^ m^2^/s in the region between 30 and 70 cm; Fig. 3B).

### Flume study—Effects of structure on seed retention

These hydrodynamic modifications produced knock‐on effects on seed retention within the structure. Once flow velocities became strong enough to resuspend seeds in the control setting, the structure had a positive influence on seed retention (*F*_1__,18_ = 13.4, *n* = 10, *P* = 0.002). In the 10‐cm/s flow environment, 100 ± 0% seed retention was observed within the structure against 90.5 ± 1.3% in the control setting. The positive effects on seed retention were strongest in the extreme environment of 55 cm/s flow (*F*_1,18_ = 10.4, *n* = 10, *P* = 0.005) amounting to a difference between 57.5 ± 2.8% and 24.3 ± 1.4% seed retention between the structure and control; Fig. 3C).

### Field study—Sediment bed level and characteristics

**Fig. 4:**
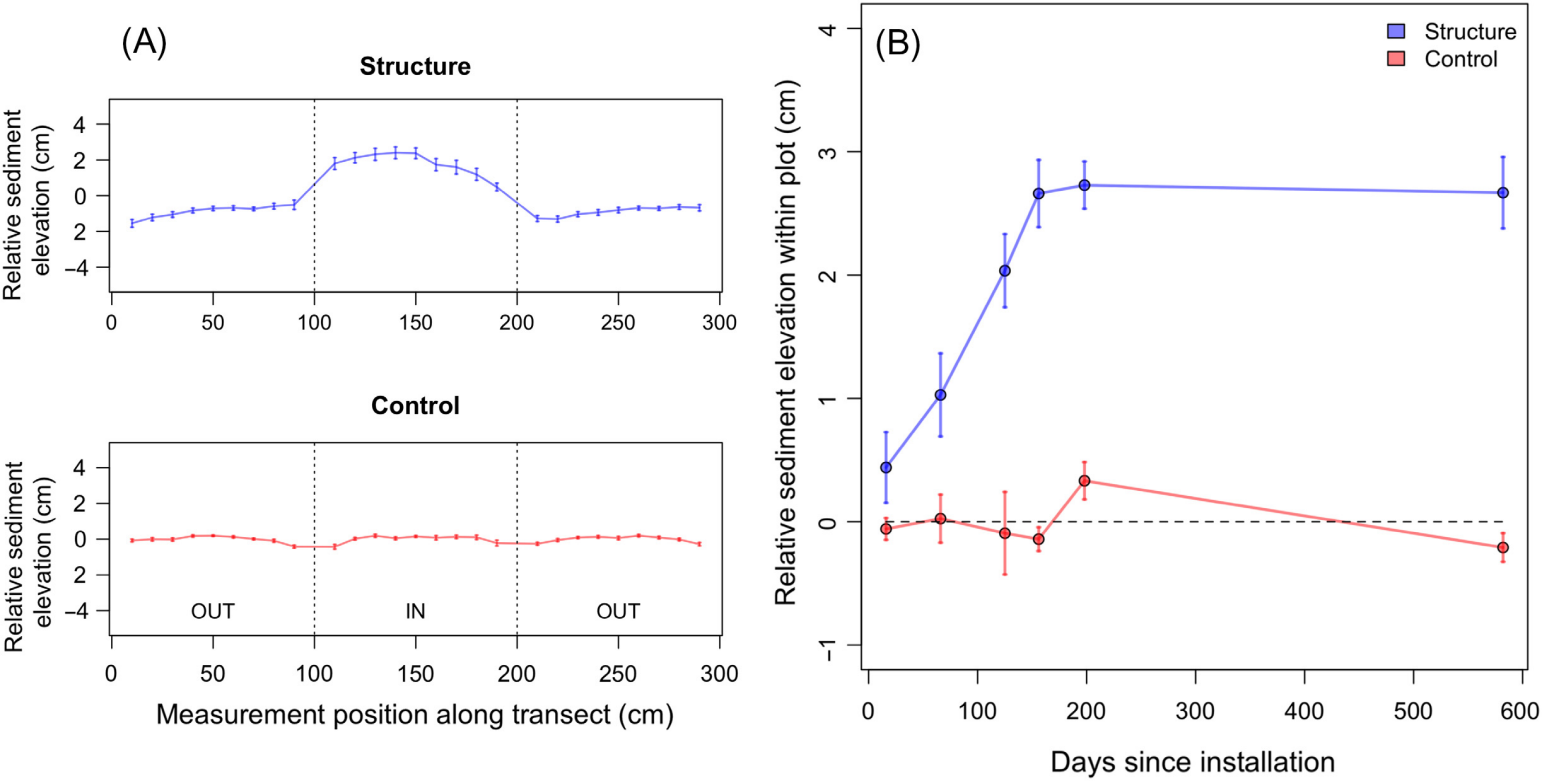
The development of sediment mounds within artificial structures (deployed in the field experiment) is contrasted against the nearby unmodified mud flat (control). (A) The 6‐cm‐tall structure produced an internal sediment mound averaging 3 cm in height. (B) Development of this sediment mound to an equilibrium state occurred over the first 200 d after installation. The relative sediment elevation was determined by subtracting each point measurement from the overall mean within the transect. Values shown here have been averaged across all replicates.

Once installed in the field, the structures caused the formation of dome‐shaped sediment platforms that developed to an average equilibrium height of 2.67 ± 0.07 cm, and an average maximum sediment height 3.3 ± 0.3 cm in the center of the structure. This represented a significant increase in sediment height when compared to the adjacent mudflat (*F*_1__,16_ = 37.63, *n* = 9, *P* < 0.0001; Fig. 4A, B). The development of the sediment platform occurred over the course of the first 200 d of the experiment and remained stable until its conclusion after 600 d (Fig. 4C). No net changes took place in the controls (*F*_1__,28_ = 2.1, *n* = 15, *P* = 0.161). Sediment sampled inside of the structures at the conclusion of the experiment showed increased silt content (*F*_1,40_ = 8.4, *n* = [structure: 14, control: 28], *P* = 0.006), from 21.17 ± 0.3% silt in the ambient sediment to 29.26 ± 0.61% silt within the structures. These shifts in sediment makeup occurred alongside changes in the nutritional content of the sediment, increasing organic carbon from 0.5 ± 0.01% to 0.7 ± 0.02% (*F*_1,40_ = 8.6, *n* = [structure: 14, control: 28], *P* = 0.006) and nitrogen from 0.053 ± 0.001% to 0.068 ± 0.002% (*F*_1,40_ = 5.6, *n* = [structure: 14, control: 28], *P* = 0.02).

### Field study—Natural recruitment

**Fig. 5:**
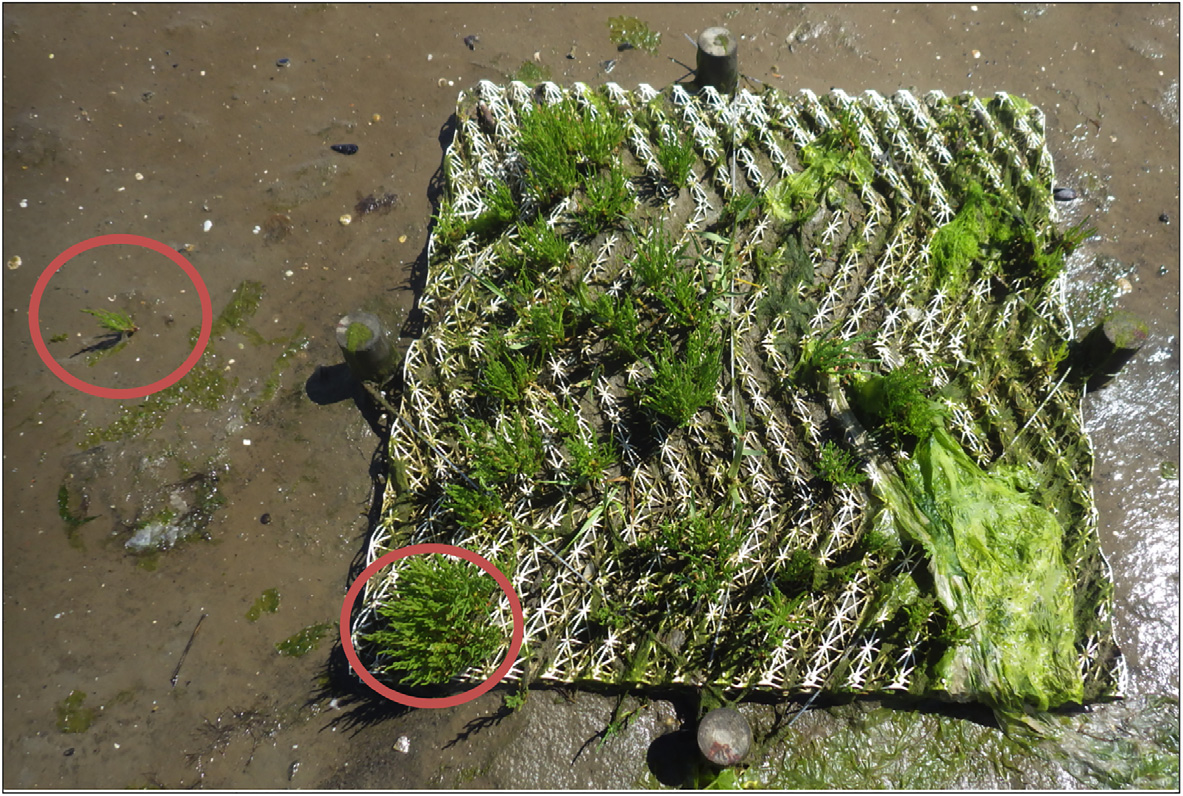
Photograph of the artificial structure used in the field experiment on Texel, the Netherlands. The structure shown here has developed a raised internal sediment platform that supports high densities of salt‐marsh recruits, predominantly *Salicornia* spp. Recruits found within the structure are also considerably larger than those found in the nearby unmodified mudflat (see individual comparison highlighting by the two red circles). Photo by Greg Fivash.

**Fig. 6:**
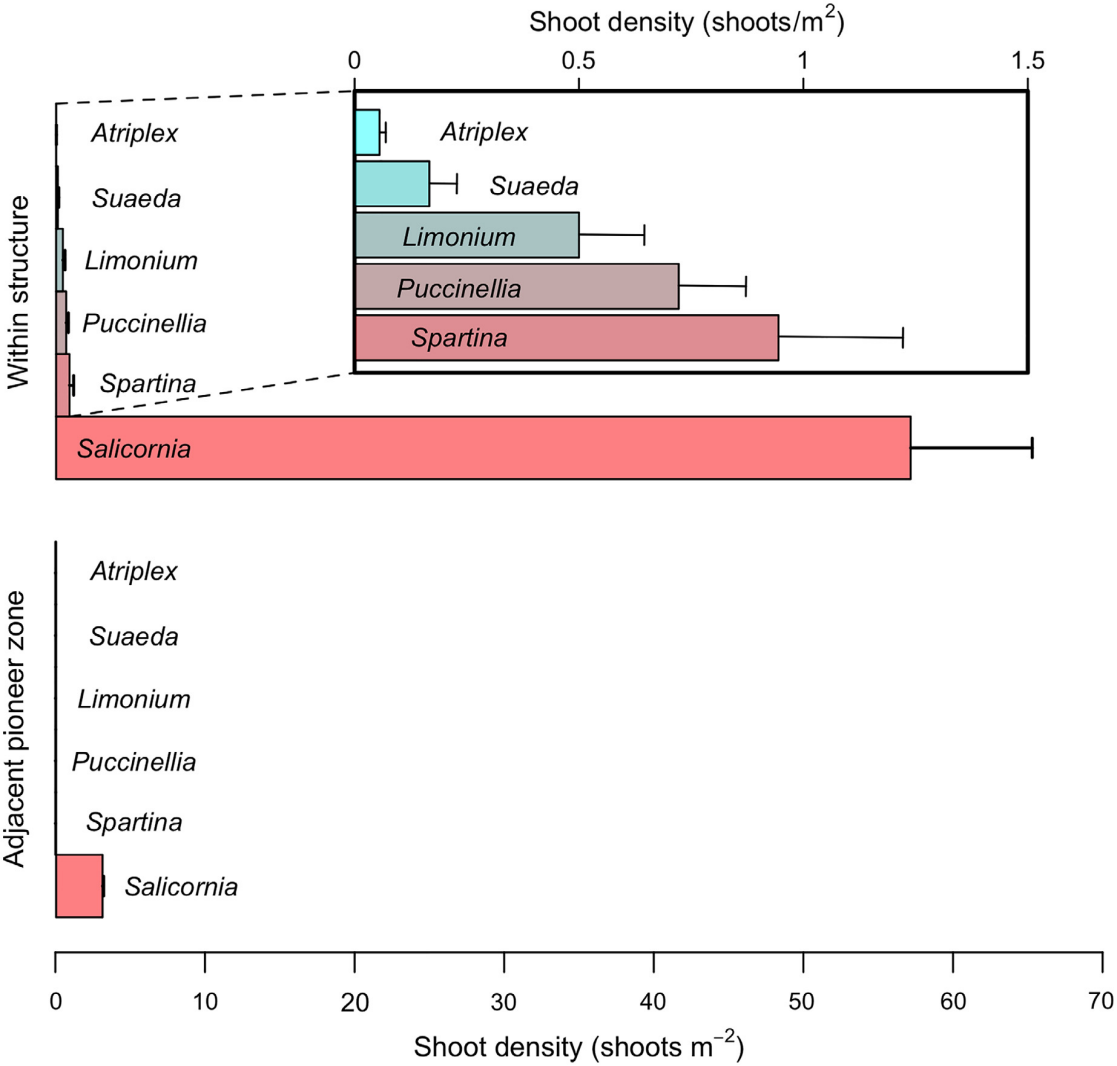
Bar plots contrast the community of seedling recruits that developed on sediment mounds created with artificial structures in the field experiment with the community on the adjacent unmodified pioneer zone (control). *Salicornia* spp., a prolific annual pioneer variety, is found within the structures at 60‐fold greater densities than in the surroundings. Although only *Salicornia* spp. was found in the unmodified pioneer zone, five other species were found to have established within the structures, including two perennial clonal pioneers (*Spartina angelica* and *Puccinellia maritima*), and three perennial mid/high‐marsh species (*Limonium vulgare*, *Suaeda maritima*, and *Atriplex portulacoides*).

**Fig. 7:**
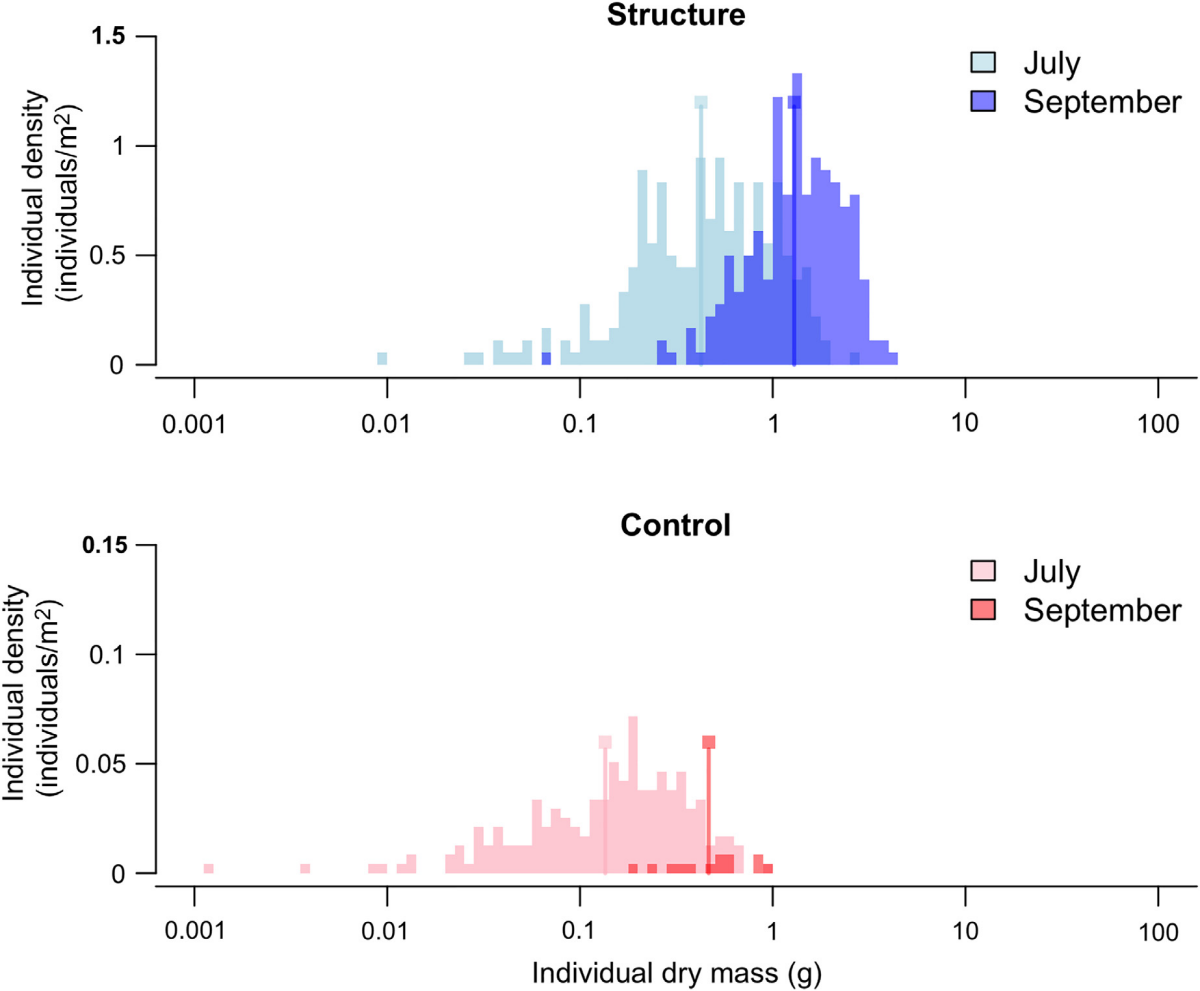
The individual density of *Salicornia* spp. is separated into size ranks to compare differences in growth and mortality between those on artificial structures (in blue) and in the unmodified pioneer zone (in pink and red, control). Repeated measurements are displayed in separate colors for July (light) and September (dark). Note the individual density scale of the control group has been magnified 10 times to ease comparisons across treatments. The line marker indicates the mean‐log average size for each group. By comparing the average size at each month, it can be seen that the artificial structures harbor faster‐growing salt‐marsh recruits. In September, very few *Salicornia* remained in the pioneer zone, whereas the individual density in the artificial structures remained similar to the density in July, suggesting a lower mortality rate in this environment.

The natural establishment of recruits was observed in the growing season of 2017 abundantly throughout our structures, and to a lesser degree in the unmodified pioneer zone (Fig. 5). These recruits were composed primarily of the annual pioneering *Salicornia* genus, but lower abundances of five other perennial species were also found exclusively within structures (Fig. 6). *Salicornia* recruits growing within the structures were both more numerous (*F*_1,508_ = 992, *n* = [structure: 18, control: 236], *P* < 0.0001) and larger (*F*_1,461_ = 166, *n* = [structure: 259, control: 204], *P* < 0.0001) than those outside of the structures. The recruits within structures also had a drastically higher rate of survival between July and September (*F*_1__,506_ = 17.2, *n* = [structure: 36, control: 472], *P* < 0.0001, visualized in Fig. 7). In July, we measured an average density of 57.17 ± 3.13 recruits/m^2^ inside the structures and 0.96 ± 0.01 recruits/m^2^ in the ambient environment. Upon our return in September, ambient densities were reduced by approximately 10 times to 0.08 ± 0.002 recruits/m^2^ while remaining high within the structures, at an average density of 43.74 ± 2.87 recruits/m^2^. In September, only 13 individual recruits remained in the 236‐m^2^ control area sampled. Recruits growing within the structures in July had an average estimated dry mass of 0.42 ± 0.01 g against 0.14 ± 0.01 g in the control plots. This difference expanded in September when plants within structures grew to an average 1.29 ± 0.01 g dry mass against 0.47 ± 0.13 g. This represented a consistent difference in size between recruits inside and outside of the experimental structures of approximately three times.

### Cross‐study comparison—Natural recruitment

Lastly, we were able to gauge differences in recruitment between naturally occurring and artificially induced mudflat heterogeneity by comparing recruitment across our observational and experimental studies. On our observational study site of Rattekaai, *Salicornia* densities averaged 8.06 ± 0.13 individuals/m^2^ and grew to reach 0.27 ± 0.003 g dry mass in July. When compared to samples from the experimental setup from the same month, we found that the artificial hummocks fostered recruits of both significantly greater size (*F*_2,__1,476_ = 60.4, *n* = [structure: 259, control: 204, Rattekaai: 1,016], *P* < 0.0001) and density (*F*_2,378_ = 4,435, *n* = [structure: 18, control: 236, Rattekaai: 126], *P* < 0.0001). Likewise, both the heterogeneous pioneer zone of Rattekaai and the artificial hummocks on Texel produced stronger recruitment and recruit growth than that found on the unmodified homogenous pioneer of Texel (Appendix S1: Fig. S5).

## Discussion

This study shows in practice how an understanding of the conditions present when natural establishment occurs can be used to develop restoration tools that invoke establishment artificially. Our observational study suggests that topographic variation in the pioneer zone of salt marshes can act as a catalyst for establishment, although it remains constrained within a fundamental niche limited in the tidal frame (Balke et al. 2016). This suggests that where such microtopography is absent from this zone, such as in our experiment study site of Texel, it is likely to be a lever by which to tip the system into a state favoring the expansion of vegetation. In our experimental study, the strong establishment of pioneer recruits within our artificial structures gathered support for the concept that promoting factors that enhance natural establishment can be highly effective in restoration application. Hence we provide support for a transition in restoration science that de‐emphasizes the role of planting and focuses more on indirect means of supporting natural establishment by addressing foundational abiotic landscape processes.

### Formation of artificially raised sediment platforms and benefits for salt marsh pioneers

The results of the experimental flume and field studies suggest that the installation of these artificial structures on the tidal flats invokes a strong, consistent reduction in the tidal flow rate within the structure. Slow flow typically enhances sedimentation rates within the low‐flow region, after an initial section of high turbulence (Bouma et al. 2007). The higher silt content observed within the structures in the field is also indicative of lower flow velocities (Bos et al. 2007). Both the observed flow and turbulence modifications, and the patterns in elevation change around the structures, are in line with descriptions by Chen et al. (2012) of how flexible vegetation typically restructures bed forms.

In our field experiment, pioneers appeared in greater densities and grew much larger on the artificial sediment platforms than elsewhere in the pioneer zone. These plants also experienced lower rates of mortality over the growing season. This may be explained as a consequence of their size, given that larger seedlings are less sensitive to hydrodynamic disturbances and sediment dynamics (Hu et al. 2015, Bouma et al. 2016). The presence of a wider composition of species on the artificial structures further indicates an environmental shift toward a lower‐stress state welcoming to a wider range of species. Of particular note are *Atriplex portulacoides*, *Suaeda maritima*, and *Limonium* *vulgare*, which are typically found on the developed marsh platform where stressors from frequent inundation, sediment mobility, and poorly developed (inorganic) soils are much weaker (Bockelmann et al. 2002, Silvestri et al. 2005, Hughes et al. 2009). The enhanced growth rates could be explained as a response to both the enhanced pore circulation within raised mounds described in Fivash et al. (2020) and as a fertilization response to the enhanced levels of nitrogen caused by the settlement of finer‐grained sediments within the structure’s low‐flow zone.

### Further means to generate microtopography

Given all of the above evidence, we argue that this study makes a strong case that emulating the microtopographic features that drive natural establishment events in the pioneer zone of salt marshes is an effective means by which to actively invoke seedling establishment. Historically, similar conditions have been created over large areas through the digging of channels into the tidal flat for the purposes of land reclamation over the Frisian coast (Beck and Airoldi 2007, de Groot and van Duin 2013). Yet the manual construction of drainages is very expensive and often impresses permanent Euclidian shapes into the landscape that do not support a fully functioning marsh (Lawrence et al. 2018). Practically speaking, the use of epibenthic structure to create microtopography by manipulating local flow rates is both an asset and a limitation. On one hand, this approach is efficient in terms of labor and materials because it makes use of tidal forces to create and reinforce microtopography. Yet it is somewhat constrained in its scalability because of the lack of long‐distance effects on topographic heterogeneity; that is, the structure does not induce changes in the sediment bed outside of the area directly confined within the structure. Another important consideration is that the scale of the epibenthic structure required to produce a sedimentary low‐flow environment will increase in environments with high current velocity. This is because strong incoming flow increases the length of the initial section of high turbulence, delaying the point at which sedimentation will begin to occur inside the structure (Bouma et al. 2007, Chen et al. 2012). Such considerations are key for this technique to be deployed effectively in the field.

Several other biological and hydrodynamic mechanisms are known that can create surface heterogeneity in bathymetry. Scale‐dependent effects caused either by flow deflection around and between epibenthic structures (Bouma et al. 2007), or locally increased soil cohesion caused by biofilm or root mat formation (Blanchard et al. 2000, Weerman et al. 2010, van de Vijsel et al. 2020) are two of the foremost biological mechanisms by which to produce topographic heterogeneity locally. The propagation of tidal channels (D’Alpaos et al. 2005) and the formation and intensification of ridge‐runnel systems on bare tidal flats may also be potentially generated through purely morphodynamic feedbacks (Williams et al. 2008, Fagherazzi and Mariotti 2012). Salt marshes themselves may be able to propagate these patterns over the pioneer zone they border by concentrating surface drainage over the tidal flat (unpublished data, Fivash GS). In addition to these natural mechanisms, the creeks in our observational study supply an example of an artificial technique, where flow was channelized through gaps in protective rock walls. Means by which to induce or make use of any one of these mechanisms to generate tidal flat heterogeneity consistently could be interesting research pathways with major applications in restoration.

### Enhancing natural recruitment processes as a major avenue for future restoration

Beyond the specific context of our model system, this study supports a general refocusing of restoration efforts away from active seed and transplantation, and toward the facilitation of natural establishment events by altering the early growth environment. Although the practice of restoration is predominantly focused on direct methods of revegetation, namely, seed addition and transplantation (Bainbridge et al. 1995, Bean et al. 2004, Abella and Newton 2009, de Groot and van Duin 2013, Mor‐Mussery et al. 2013, Han et al. 2015), simple methods have occasionally been found that can dramatically enhance natural recruitment indirectly. One major example has been the installation of permeable dams to induce mangrove establishment (Kamali and Hashim 2011). Larger‐scale hydrological management, when possible, has also been a successful approach that relies on natural recruitment (Lewis and Streever 2000). In arid systems, for instance, the installment of temporary irrigation systems to promote natural establishment of higher successional states in degraded environments has been used to great effect in the American southwest for decades (Bainbridge et al. 1995). However, the successful manipulation of many other key environmental factors for restoration purposes is not yet widespread. For instance, the development of rehabilitation strategies for biological soil crusts and other means by which to mimic their beneficial activity to promote vegetation development in drylands has not yet been realized at a field scale (to the knowledge of the authors), although research is ongoing (Doherty et al. 2015, Chock et al. 2019). This is despite the fact that limiting factors controlling the establishment of these crusts and the vegetation they promote are well known (Bowker 2007 and those referenced therein). Given the advancing state of knowledge about natural recruitment processes, it seems likely that many more efficient and scalable means of restoration should reveal themselves in due course.

## Conclusion

Natural establishment within degraded landscapes is the mechanism by which ecosystems expand and reverse degradation naturally. The widespread use of transplantation techniques to restore hostile, physically driven degraded landscapes has been an effective tool when other possibilities have not been available. Yet, the poorer performance of these techniques in disturbance‐driven systems often means that their scalability is limited to the recovery of small areas. A switch from these older techniques to those that may address much more ambitious project scales through the induction of natural recruitment would represent the next stage in the evolution of restoration ecology, as long as studies can continue to demonstrate that such forces can be consistently controlled. The discovery of new means to promote establishment and the honing of these techniques will no doubt be valuable tools by which to combat the ongoing degradation of natural systems around the world.

## Supporting information

AppendixS1

## Data Availability

Data from this paper and the scripts used to perform the analysis (Fivash et al. 2021) are archived and publicly available within 4TU.Research Data.F https://doi.org/10.4121/13365323
