# Supplementary material for: Restoration of biogeomorphic systems by creating windows of opportunity to support natural establishment processes
Source: Ecol Appl. 2021 May 7;31(5):e02333. doi: 10.1002/eap.2333 (PMC8365657; doi:10.1002/eap.2333)
Supplement: Supplementary file 1 — AppendixS1 [file EAP-31-e02333-s001.pdf]

Supporting Information. Fivash, G.S., R.J.M. Temmink, M. D'Angelo, J. van Dalen, W. Lengkeek, K. Didderen, F. Ballio, T. van der Heide, and T.J. Bouma. 2021. Restoration of biogeomorphic systems by creating windows of opportunity to support natural establishment processes. Ecological Applications.

Appendix S1: Supplementary Figures

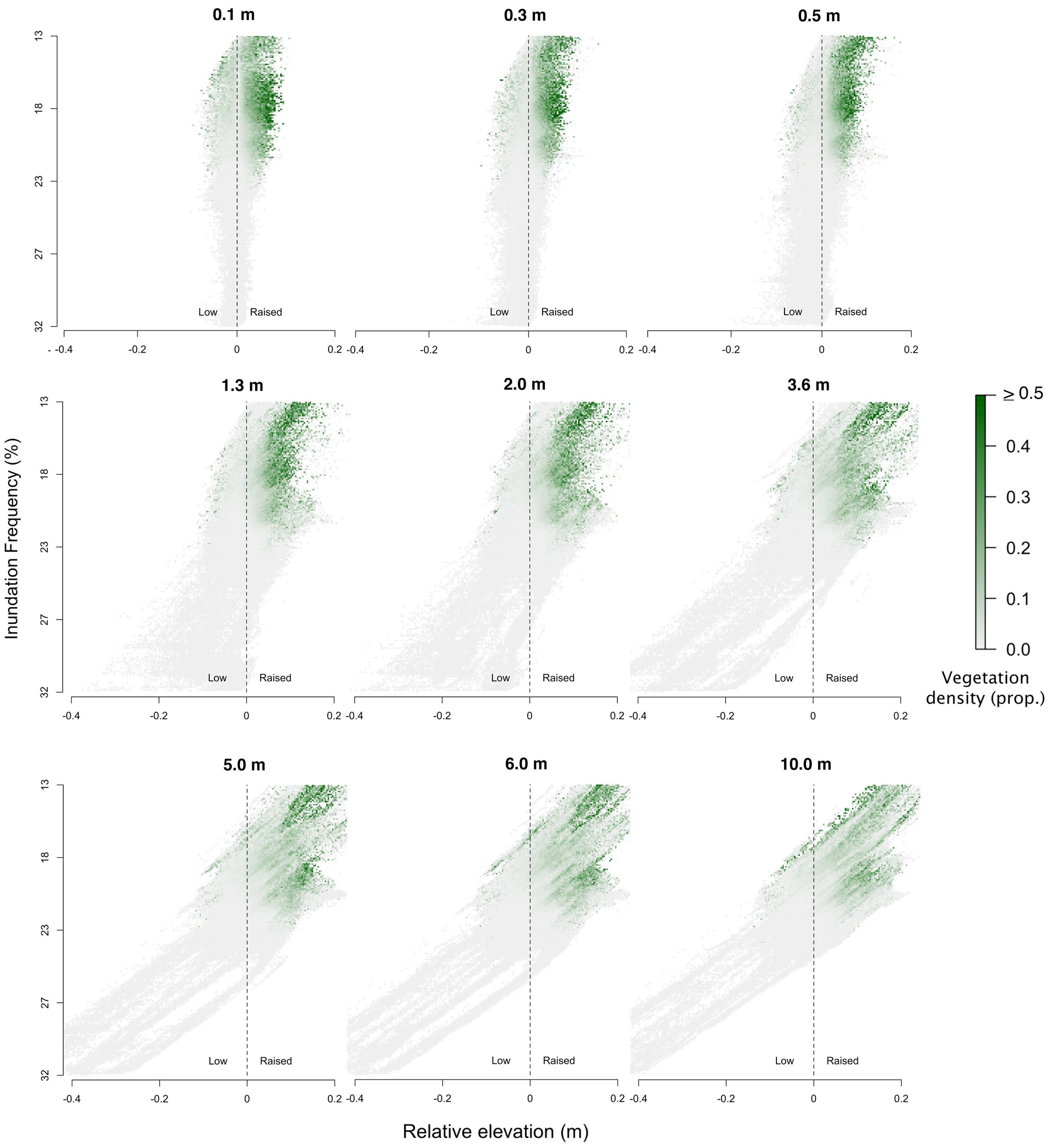

Fig. S1. An array of two-dimensional density plots is displayed to demonstrate how changes in the scale at which the relative elevation is calculated, affect its correlation with vegetation density in the observational survey. Above each panel the scale of measurement is displayed in bold font. The correlation between high vegetation density and raised relative elevation remains consistent across all spatial scales. As the scale of measurement increases the correlation between the inundation frequency and relative inundation also increases, visible as development of diagonal slope in the measured values (grey-green color band).

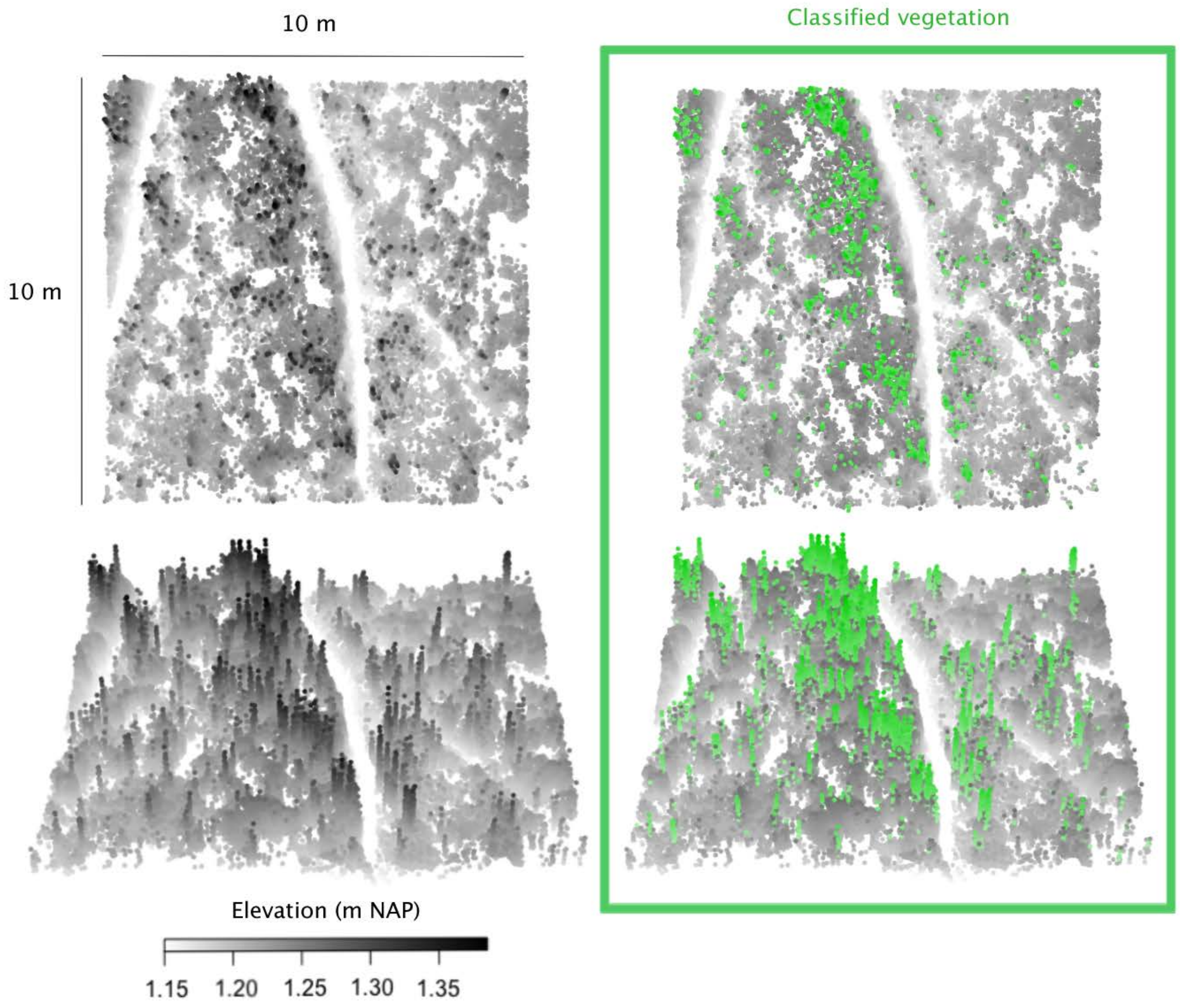

Fig. S2. A visualization of the method for vegetation classification from the raw laser scan point cloud used in the observational survey. Here, a 10 m^2 frame of the point cloud is displayed from a top-down and angled orientation. Grey tones from light to dark indicate increasing elevation (left). The presence of vegetation is notable as vertical stacks of points protruding from the mudflat. These vertically aligned points are isolated based on their slope relationship with neighboring points (see text for details). Points that have been classified as vegetation (right) are displayed in green. Since distinguishing vegetation individuals is both challenging and unreliable when vegetation congregates in tight clusters, we measure vegetation density instead as the proportion of points within an area that have been classified as vegetation, a value bounded between 0 and 1.

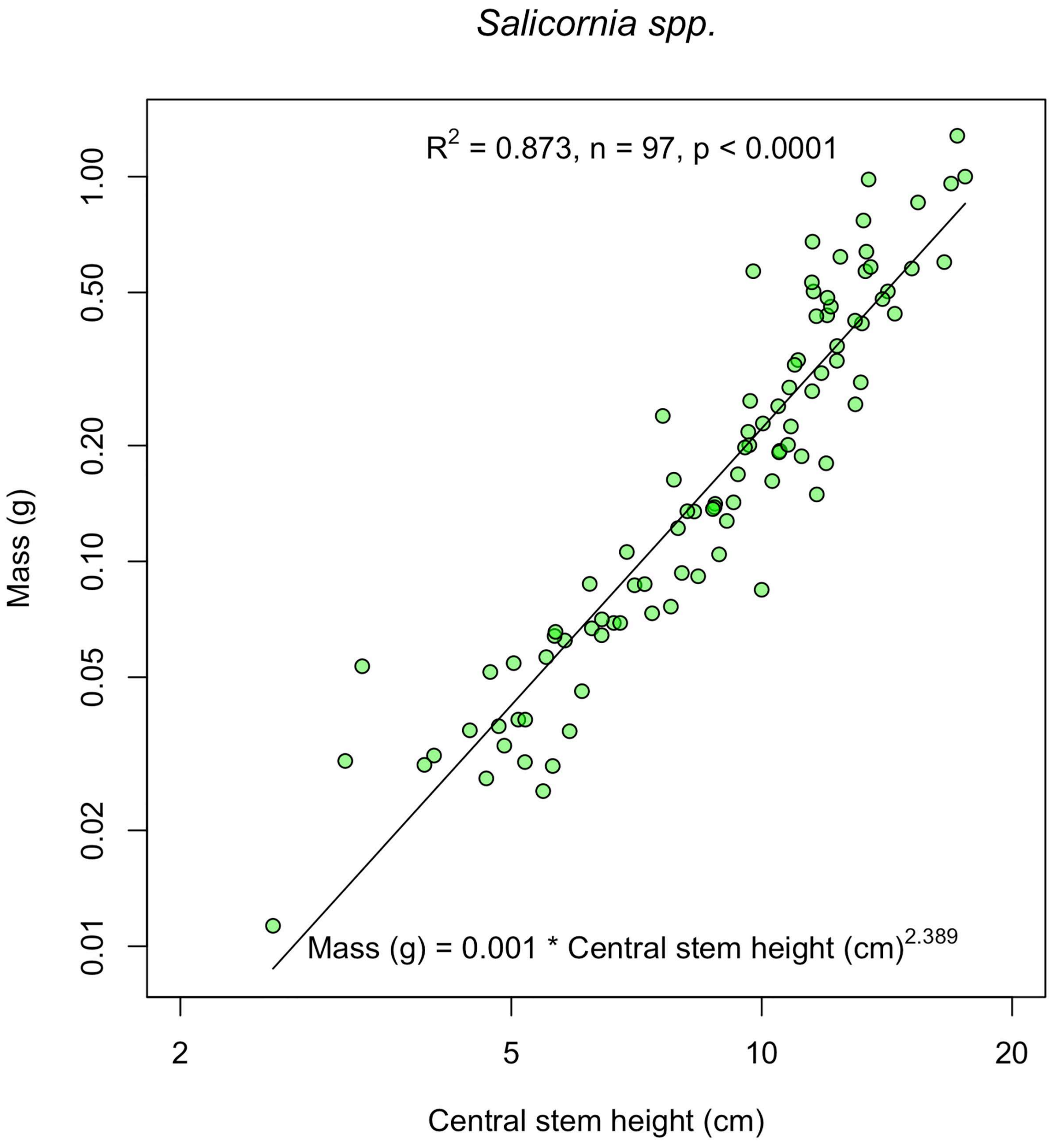

Fig. S3. The calibration curve used to calculate *Salicornia spp.* aboveground dry biomass from (non-destructive) field measurements of the maximum height of an individual plant.

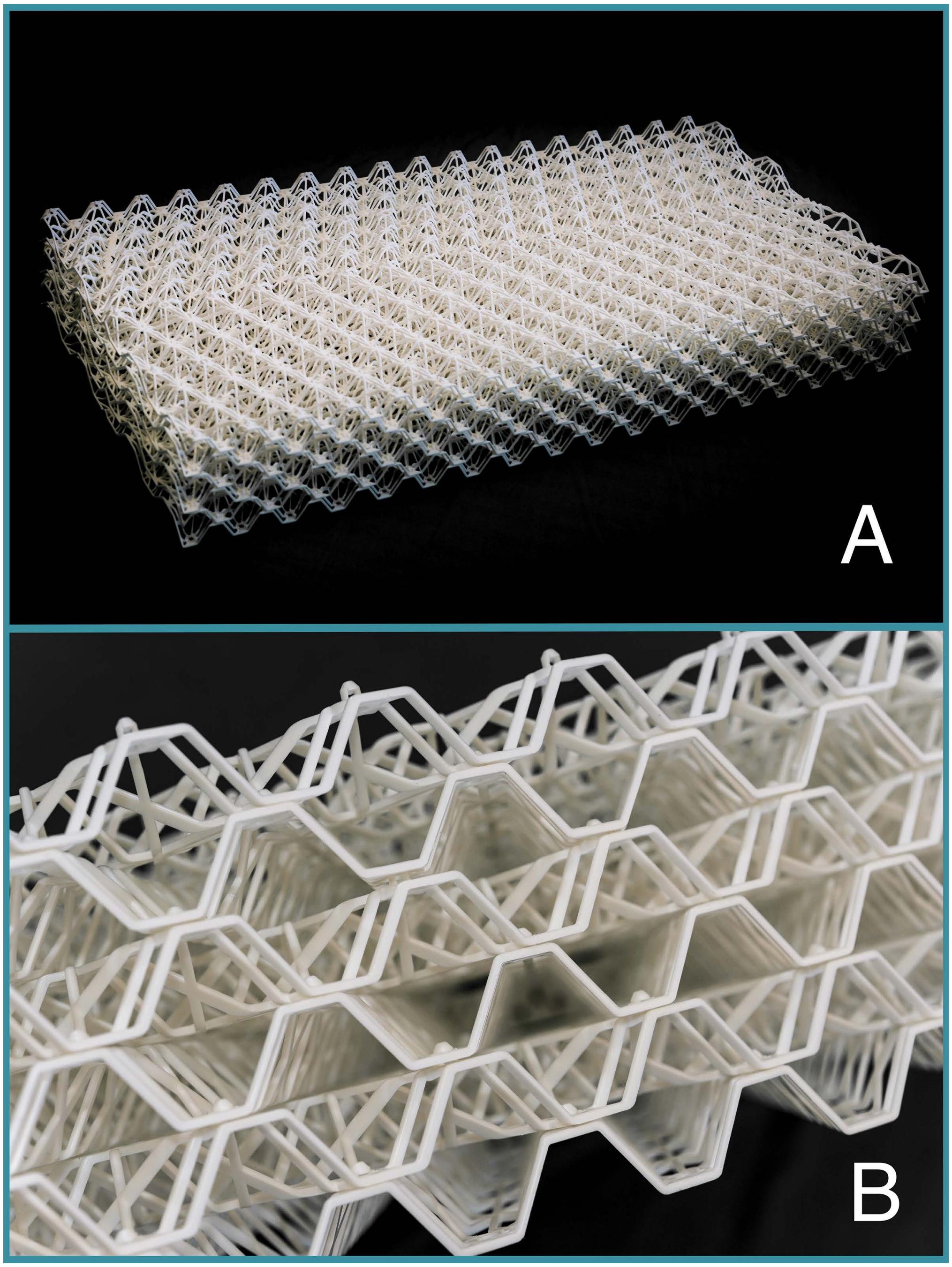

Fig. S4. Photos display BESE-elements, the biodegradable artificial structure used to suppress tidal flow velocity and generate raised sediment mounds on the tidal flat in this study. Photo perspectives: (a) overview of structure, (b) lateral view, showcasing the pore-size and internal complexity. Photo credit: Udo van Dongen, Bureau Waardenburg.

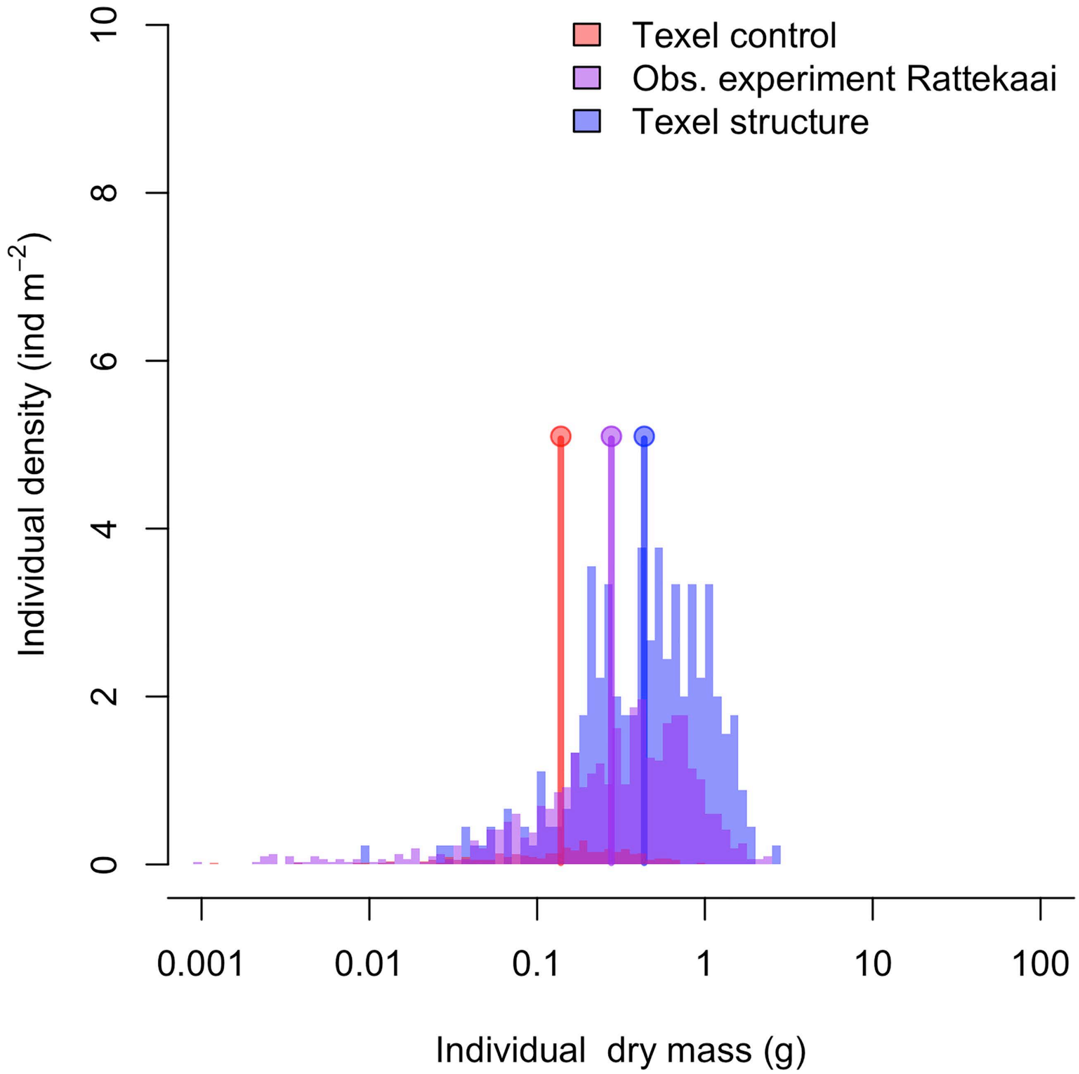

Fig. S5. The individual density of *Salicornia* spp., divided amongst size classes, is used to compare seedling establishment between the observational and experimental studies. The marker lines indicate the mean-log average size of each group. In the experimental study site on Texel, which lacks micro-topographic features, average recruit size and recruitment densities were lower than those found on the micro-topographically complex site of the observational study, Rattekaai. By implementing artificial structures to generate raised sediment mounds on Texel, recruitment was strongly facilitated such that both recruit densities and recruit sizes, originally very low, became even greater than those found on Rattekaai.
